# A Precessing-Coin-like Rotary Actuator for Distal Endoscope Scanners: Proof-of-Concept Study

**DOI:** 10.3390/mi16010111

**Published:** 2025-01-20

**Authors:** Nirvana Gharib, Mohammad Reza Yousefi Darestani, Kenichi Takahata

**Affiliations:** 1 Department of Electrical and Computer Engineering, University of British Columbia, Vancouver, BC V6T 1Z4, Canada; 2 School of Biomedical Engineering, University of British Columbia, Vancouver, BC V6T 1Z3, Canada

**Keywords:** optical scanner, electromagnetic actuator, precessional rotation, medical imaging, optical coherence tomography, Raman spectroscopy

## Abstract

This paper presents, for the first time, a rotary actuator functionalized by an inclined disc rotor that serves as a distal optical scanner for endoscopic probes, enabling side-viewing endoscopy in luminal organs using different imaging/analytic modalities such as optical coherence tomography and Raman spectroscopy. This scanner uses a magnetic rotor designed to have a mirror surface on its backside, being electromagnetically driven to roll around the cone-shaped hollow base to create a motion just like a precessing coin. An optical probing beam directed from the probe’s optic fiber is passed through the hollow cone to be incident and bent on the back mirror of the rotating inclined rotor, circulating the probing beam around the scanner for full 360° sideway imaging. This new scanner architecture removes the need for a separate prism mirror and holding mechanics to drastically simplify the scanner design and thus, potentially enhancing device miniaturization and reliability. The first proof-of-concept is developed using 3D printing and experimentally analyzed to reveal the ability of both angular stepping at 45° and high-speed rotation up to 1500 rpm within the biologically safe temperature range, a key function for multimodal imaging. Preliminary optical testing demonstrates continuous circumferential scanning of the laser beam with no blind spot caused by power leads to the actuator. The results indicate the fundamental feasibility of the developed actuator as an endoscopic distal scanner, a significant step to further development toward advancing optical endoscope technology.

## 1. Introduction

Endoscopic devices play critical roles in many fields of today’s medicine. Their use is highly effective and nowadays essential in diagnosing medical conditions, while enabling physicians to access various areas inside the body, ranging from arteries and the esophagus to the peritoneum and abdominal tissues [1,2,3]. These endoscopic imaging capabilities enhance early detection of lesions such as cancers, a key to accurate and timely diagnosis and treatment, forming the basis for not only effective intervention strategies in healthcare practice but also contributing to pathologic studies [4,5]. While commonly associated with gastrointestinal disorders and cancer screening, endoscopy has applications beyond oncology, including diagnosis and management of cardiac problems [6,7]. Although they are frequently used for visualizing surfaces like the interior walls of organs such as the stomach or colon, they can also be equipped with additional tools, such as ultrasound, for procedures like biopsies or surgery [8,9,10,11]. Advanced endoscopic techniques, such as confocal laser endomicroscopy or optical coherence tomography (OCT), can offer detailed imaging of tissue layers beneath the surface. Therefore, while endoscopic imaging primarily focuses on characterization of surface morphologies of target tissue, it can provide insights into structures of a surface layer [10,12,13,14,15]. Different imaging modalities, such as OCT, Raman Spectroscopy (RS), and ultrasound, can be combined with endoscopes for more accurate diagnosis [16]. Multimodal endoscopy that leverages different imaging techniques is a promising approach to enhancing physicians’ ability for lesion diagnosis. For example, a combination of OCT and RS allows for optically and chemically distinguishing between healthy and cancerous tissues with high accuracy [17,18].

Enhancing the endoscopic field of view (FOV) by enabling 360° imaging can significantly improve the endoscope’s capability and related examination process. Toward this end, there have been a wide range of efforts, including applications of micro-electro-mechanical systems (MEMS) technology, which report distal optical scanners for endoscopes to realize their 3-dimensional (3D) imaging [19,20,21,22,23,24]. MEMS scanners with integrated micromirrors are typically limited in their FOV (ranging 25–150° [1,25]) due to their nature of planar architectures. Moreover, packaging these planar scanners at the distal end of endoscopic devices poses challenges in achieving a reliable and cost-effective solution. A range of studies have developed miniaturized distal scanners that rely on the rotation of a small prism mirror using a micromotor, allowing for fully circumferential 360° scanning of a probing light [26,27,28,29,30]. Many of these scanners used commercial small motors, which not only have bulky and long bodies (partly due to needing a gear box) but also are highly expensive (costing between USD1000 and USD2000) [28,31,32]. Besides, they offer no ability to control low-speed or stepping actuation required for certain endoscopic techniques like RS. One main challenge in realizing practical micromotors is the bearing that provides low friction in a miniaturized form [33,34]. To address this challenge, the electromagnetic rotary actuators based on the use of a ferrofluid bearing were developed [35,36]. The ferrofluid is a type of smart fluid that moves towards areas with higher magnetic fields, and when applied to a permanent magnet, accumulates at its poles to create a self-sustained lubricant layer that passively levitates it, enabling frictionless movement of the magnet rotor/slider with no power consumption. While this ferrofluid approach significantly simplified the bearing design that contributed to the actuator miniaturization, these actuators had to use a drive shaft to transfer the rotor’s motion to the prism mirror for its rotation, primarily in order to avoid its contamination with the ferrofluid, and therefore, their axial lengths inherently become large (>5 mm), to limit the flexibility of the probe tip when integrated. The use of the drive shaft for the rotor-mirror assembly can also complicate the manufacturing process and thus potentially increase the product cost.

**Figure 1:**
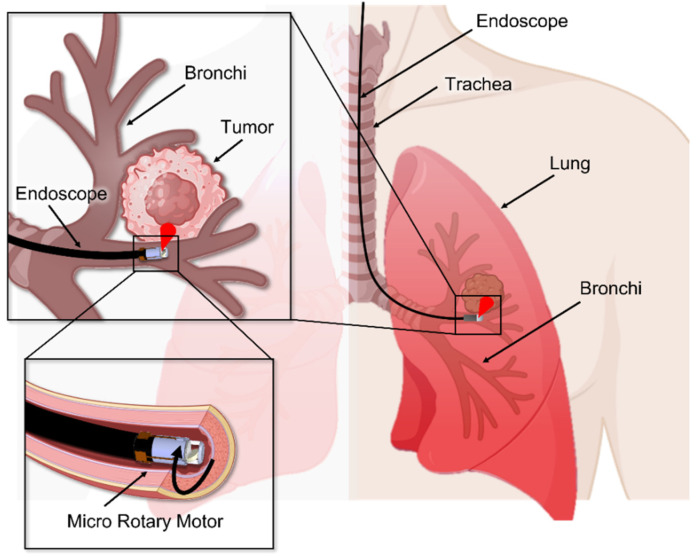
Conceptual diagram illustrating the distal 360° optical scanner with the new rotary actuator embedded at the tip of an endoscopic probe, highlighting its potential application example for peripheral lung diagnosis.

This work explores a first-of-its-kind rotary actuator based on an extremely simple and small-length design for the application to endoscopic distal scanners compatible with multimodal imaging. This actuator/scanner uses an angled disc magnet as the rotor, which is made to have a reflective surface on the backside, serving as a mirror to circumferentially scan a probing light when electromagnetically rotated. The disc rotor is designed to roll around the cone-shaped base to establish a motion similar to spinning-coin precession. This unique design removes the need for the components of the prism mirror and drive shaft that were essential in the preceding designs [35,36]. The primary design goal here is to reduce the axial length of the scanner, which leads to a shorter rigid portion at the distal end of the probe that enhances its flexibility and maneuverability, particularly in tight and/or curvilinear lumens, thereby reducing the potential for tissue damage while enhancing the reliability of the device by using a minimal number of moving mechanical components. The entire actuator is designed to have a hollow structure, allowing an optical beam to pass through the actuator body for bending at the tilted mirror surface of the rotor, eliminating light blockage and shadows caused by power leads to the actuator [37]. This scanner architecture is aimed to offer a 360° FOV for both high-speed rotation and angular stepping modes of imaging without blind spots, making it suitable for multimodal imaging applications including OCT and RS [13,18]. Figure 1 illustrates a potential application for lung cancer diagnosis using an endoscope device enabled with the integrated distal scanner studied in this work. The main objective is to explore the feasibility of the proposed scanner design experimentally and verify the concept using the first proof-of-concept prototype. While the design focus is placed on this objective, rather than miniaturization, it is configured to ease downsizing for the next step. The following section provides an in-depth description of the working principle and design of the developed scanner (Section 2) and prototyping process and results for the aforementioned device (Section 3). This is followed by the experimental results that characterize the actuator’s performance and then show its optical scanning capability using a preliminary test model (Section 4).

## 2. Working Principle and Design

**Figure 2:**
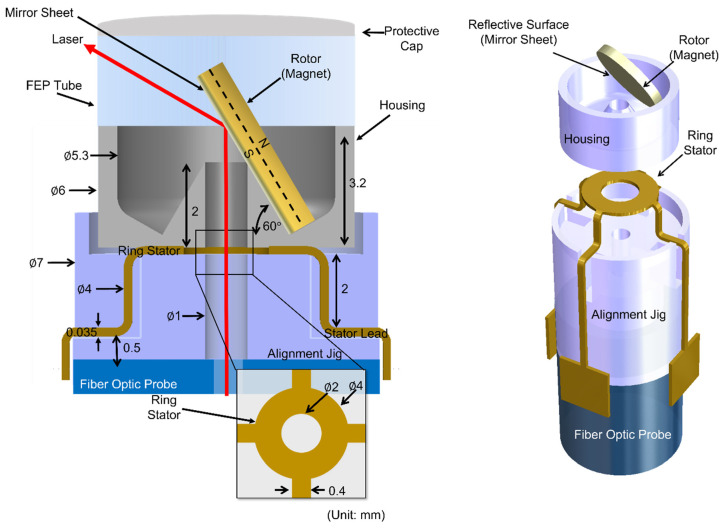
Schematic cross-section of the design (**left**) and exploded 3D view (**right**) of the scanner device investigated.

The overall design of the proposed endoscopic scanner is illustrated in Figure 2. The rotary actuator consists of a permanent disc magnet polarized along its thickness that serves as the rotor, and a planar single-layer ring stator positioned beneath the rotor. The disc rotor is enclosed in a housing that physically supports it in an inclined position (at 60° in the current design) with a cone-like structure on which the rotor is forced to roll around due to electromagnetic fields with specific orientations set by the underneath stator. The rotor’s cone-shaped base has a through hole in its center, serving as the path of an optical beam supplied from the fiber-optic endoscope coupled with the scanner. The ring stator is positioned on its hollow custom jig, designed to align with the housing and rotor’s base. The optical beam that passes the through holes of the stator and the rotor’s base reflects off the back of the rotor, which is designed to be optically reflective. Consequently, the beam bent on the back of the inclined rotor is circulated around the distal end of the endoscope as the scanner drives the rotor, and the scattered light reflected on the tissue being imaged is collected back through the same path of the scanner. Due to the magnet’s inherent surface roughness and inability to serve as a reflective surface, a thin mirror sheet (shaped to match the rotor size) is laminated onto the corresponding side of the disc magnet in the current design.

**Figure 3:**
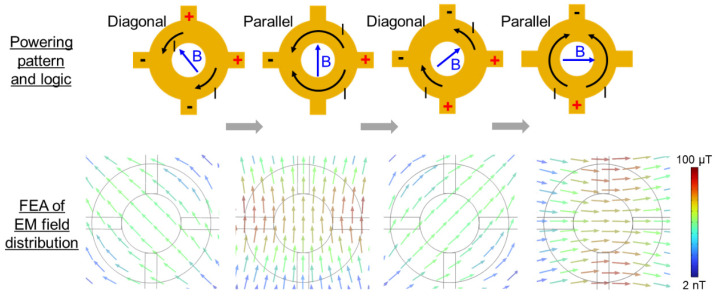
Ring-stator powering logic for actuation (**top**), depicting four displacement steps that provide 135° rotation with 45° angular stepping; the symbols +/− indicate the polarities of applied voltage that create the drive currents, *I*, flowing through the ring stator in either the diagonal or parallel pattern, resulting in an electromagnetic field, *B*, with varying orientations that displace the inclined disc magnet located above. Simulated electromagnetic fields produced at the illustrated logic steps (**bottom**).

The ring stator incorporates four input leads that are digitally activated with either a positive or negative voltage to induce different patterns of current flow within the stator loop, generating electromagnetic fields that define the rotor’s orientations [35]. Two field patterns, namely diagonal (set by two positive and two negative voltages) and parallel (set by one positive and one negative voltages), are employed with the stator to create a rotation in eight 45° displacements as illustrated in Figure 3 (showing a partial rotation only with four steps and their corresponding input logic). This figure also shows the resultant electromagnetic fields over the stator (3 mm above the stator plane) obtained via finite element analysis (FEA), illustrating how these diagonal and parallel patterns create the driving fields. When these steps are run sequentially, they result in a full rotation of the inclined disc magnet. The rotational speed is controlled by setting the interval between the adjacent steps. This principle enables the scanner to perform both angular stepping and continuous high-speed actuations by varying the frequency of signal switching, offering the compatibility with different endoscope modalities.

## 3. Proof-of-Concept Prototyping

### 3.1. Scanner Device

**Figure 4:**
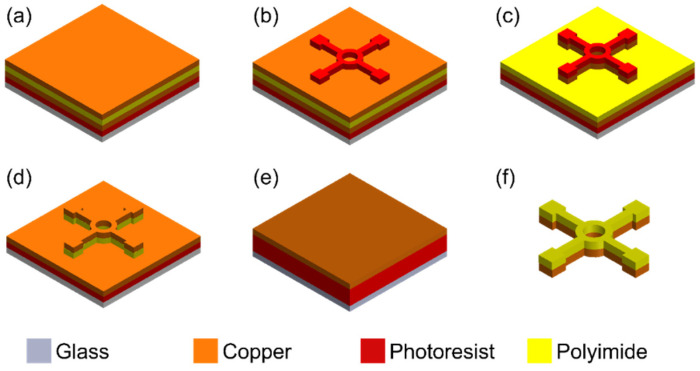
Fabrication process of the ring stator with powering leads: (**a**) Laminating the double-sided copper-clad polyimide film onto the glass wafer; (**b**) spin-coating a photoresist and lithographic patterning; (**c**) wet etching the exposed front copper clad; (**d**) wet etching the exposed polyimide; (**e**) laminating the film to a glass wafer upside down; (**f**) wet etching the backside copper clad layer away and dissolving the photoresist to release the individual stator structures from the wafer.

**Figure 5:**
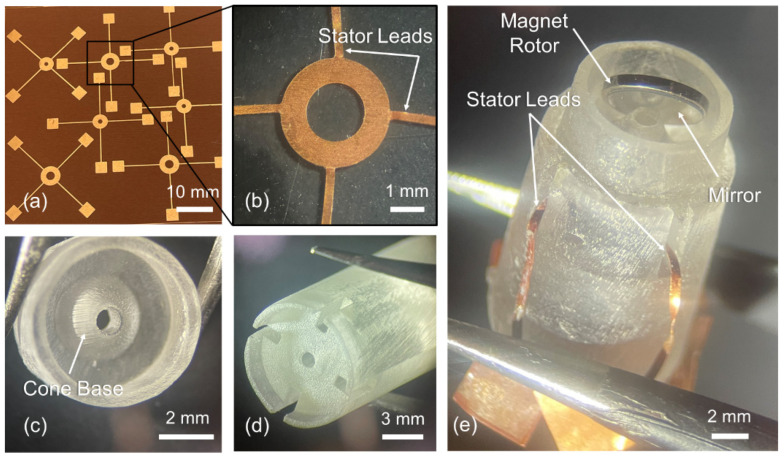
Fabrication results: (**a**) Top view of multiple ring stators (showing a sample after patterning the copper layer); (**b**) released stator structure; (**c**) scanner’s 3D-printed housing; (**d**) alignment jig; (**e**) assembled scanner prototype.

The stator circuit made of a copper layer, featuring a 4-/2-mm outer/inner diameter ring with four 400-μm-wide powering leads, is patterned using double-sided copper-clad polyimide sheet (AP9111R, DuPont, DE, USA, 35-μm-thick copper with 25-μm-thick polyimide) through a microfabrication process based on a combination of photolithography and wet etching (Figure 4); details of the process steps are discussed somewhere else [35]. Briefly, the stator-circuit patterns are lithographically printed onto a photoresist that has been spin-coated on a copper-clad substrate. These patterns are then transferred to the copper layer using a wet etchant (Figure 5a). The excess polyimide not covered by the copper patterns is etched away to isolate the fully patterned stators from the original polyimide sheet. The backside copper layer and the photoresist are removed to release the completed stator structures (Figure 5b). The housing and stator jig components (Figure 5c,d) are fabricated using stereolithography 3D printing (Form 3+ with clear V4 resin, Formlabs, MA, USA). After 3D printing, the components are washed in isopropyl alcohol and then in deionized water, followed by curing at 60 °C using the custom unit of the printer (Form Cure, Formlabs, MA, USA) for 1 h to stabilize and strengthen the polymerized parts. A neodymium disc magnet (D1021A, SuperMagnetMan, AL, USA) with a diameter of 4 mm and a thickness of 0.5 mm serves as the rotor in this prototype. To achieve a high optical reflectivity on one side, a 100-μm-thick self-adhesive acrylic mirror film is laser-cut (using IX-280-ML, IPG Photonics, MA, USA) and laminated onto the above magnet (Figure 5e).

The scanner prototype is built up by assembling the prepared components (Figure 5c); the ring stator is placed on its jig component and then the housing is press-fitted into the jig. The four leads of the flexible stator are bent downward (Figure 5e). The rotor is held inside the housing in a way to sit on the hollow cone base (Figure 5c). This proof-of-concept prototype does not incorporate the protective cap and transparent window walls illustrated in Figure 2, as they are not essential for testing its fundamental scanning performance, which is the focus of the current study.

### 3.2. Actuator Controller

A drive circuit for the scanner is established around a programmable microcontroller board (Arduino Uno, Arduino, MA, USA). This microcontroller is used to set the polarity of the voltage supplied to each of the four stator leads via a complementary-metal-oxide-semiconductor inverter circuit (IRFU9024NPBF and IRLU024NPBF, Infineon Technologies AG, Neubiberg, Germany) connected to each lead. All four inverters are powered by an external DC power supply (1902B, BK Precision, CA, USA) to provide the currents required to drive the actuator. For both the diagonal and parallel configurations, the positive and negative voltages are digitally coded as 5 V and 0 V, respectively. In the parallel configuration, ideally, the two leads except for those positive/negative leads are electrically floated to allow for the intended current flow in the ring. Since this particular setting is not available with the current system, alternatively, these two leads are set to hold a mid-level voltage (2.5 V), encoded as a 50% duty cycle of 5-V square pulses at a 490-Hz frequency in the microcontroller (which, after being transferred through the wire, produce an averaged DC signal of 2.5 V due to the wire’s parasitic capacitance), which provides the desired current for the parallel configuration. The abovementioned powering scheme is applied to both stepping and high-speed actuation of the scanner.

## 4. Results

The electromechanical performance of the prototyped device was experimentally tested and analyzed. The results from the stepping-mode actuation and high-speed operation of the device are presented in Section 4.1 and Section 4.2, respectively, followed by the thermal behavior of the operating device in Section 4.3. Preliminary testing of circumferential optical scanning was also conducted by directing a laser beam into the scanner, demonstrating the continuous high-speed rotation of the laser as a proof-of-concept in Section 4.4.

### 4.1. Stepping-Mode Test

**Figure 6:**
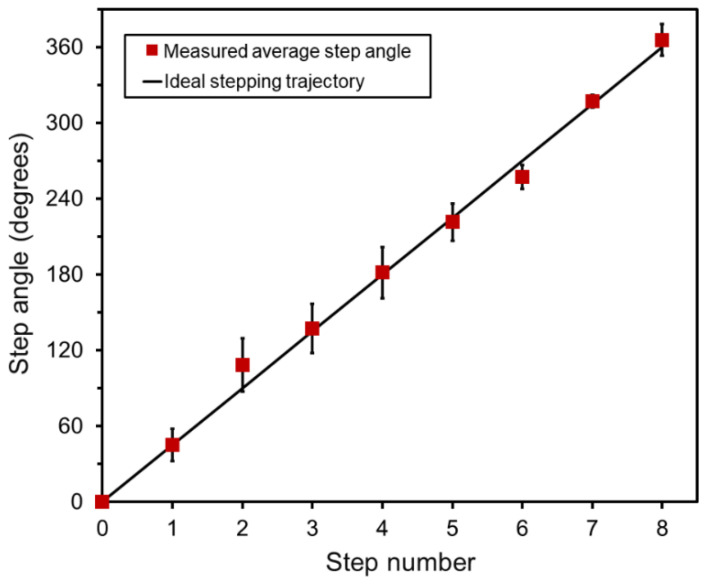
Angular displacements measured using the fabricated prototype (error bars represent ± standard deviation (SD)).

The angular actuation of the scanner was tested with the 8-step sequence (Figure 3) that theoretically provided 45° stepping. This test involved the actuator stopping at each step for 1 s, and a digital image was captured at each step. To precisely measure the angular displacement, a protractor was placed beneath the scanner, allowing for accurate readings of the angle change at each step. The measurement was repeated seven times. The results are plotted in Figure 6, showing the absolute average angles measured at each step of one rotation, alongside the ideal angular trajectory. As can be seen, the measurement showed consistent angular motions, with an average angular error of less than 4° with respect to the theoretical case.

### 4.2. High-Speed-Mode Test

**Figure 7:**
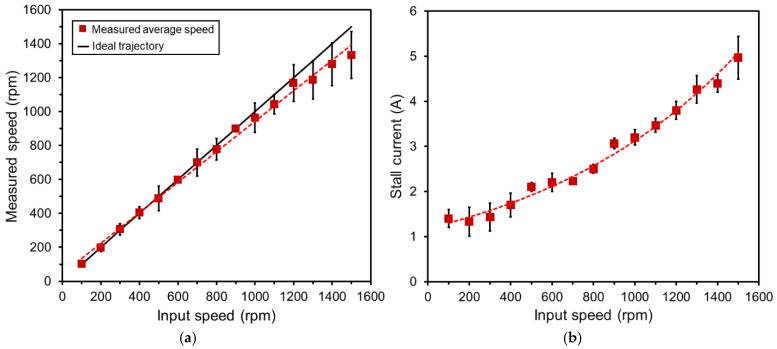
Characterization results from high-speed actuation tests: (**a**) Measured rotational speed (shown with ± SD) vs. programmed speed; (**b**) average stall current (shown with ± SD) required to drive the rotor at specific speeds.

The scanner was tested for various programmed speeds up to 1500 rpm, the maximum level that the actuator maintained uniform and stable rotation with the current design. Through visual observation, the scanner appeared to provide consistent increases in rotation speed according to the programmed speed. To assess the actual output speeds produced with respect to the input ones, the dynamic behavior of the rotor was recorded using a high-speed camera (i-SPEED LT, Olympus, MA, USA) and a real rotation rate was extracted from the continuously captured images. Figure 7a illustrates the average of the actual rotation speeds measured in seven repeated tests as a function of the input speed set in the programmed code. It can be observed that the actual speed closely matched the input speed for the range up to approximately 1000 rpm, beyond which small deviations became noticeable. The most likely reason for this difference is that dynamic friction between the magnet’s surface and the cone base increased and started to show its effect in reducing the speed when it reached that level. This potential source can be explained with the electromagnetic force acting on the inclined rotor, which attracts the rotor toward the cone base; therefore, raising the speed or the drive current increases the attractive force and thus the friction between the rotor and the cone base as well. The total currents required to drive the rotor at target speeds were also recorded and plotted in Figure 7b. As shown, the initial current needed to initiate the rotation was 1.5 A, which increased to 5 A to achieve a rotation speed of 1500 rpm. The probable reason for the need to increase the current is that the torque, which rises with the current, must be increased for the rotor to catch up with the speed of current switching.

### 4.3. Thermal Characterization

For endoscope scanners, the operating temperature is a critical factor for their medical use. The current flow through the stator causes Joule heating, resulting in an increase in the scanner’s operational temperature [35,36], which limits the rotational speeds within a specific range to ensure safety for in-vivo operation. In particular, the surface temperature should not exceed ~45 °C to avoid any potential damage to the surrounding tissue in contact with the device [38]. To assess the thermal behavior of the fabricated prototype, an infrared camera (VarioCam HiRes 1.2M, Jenoptik AG, Jena, Germany) was used to capture temperature maps of the operating device while raising the speed (and the drive current) of the scanner. The scanner was operated for 2 min for a given drive current to ensure the temperature was saturated and stabilized. The measurement was repeated three times to capture the average of the maximum temperature on the exterior surfaces of the scanner, which was on the power leads to the stator. To evaluate the Joule heating effect itself, the same measurement was performed without the rotor in the device as well.

**Figure 8:**
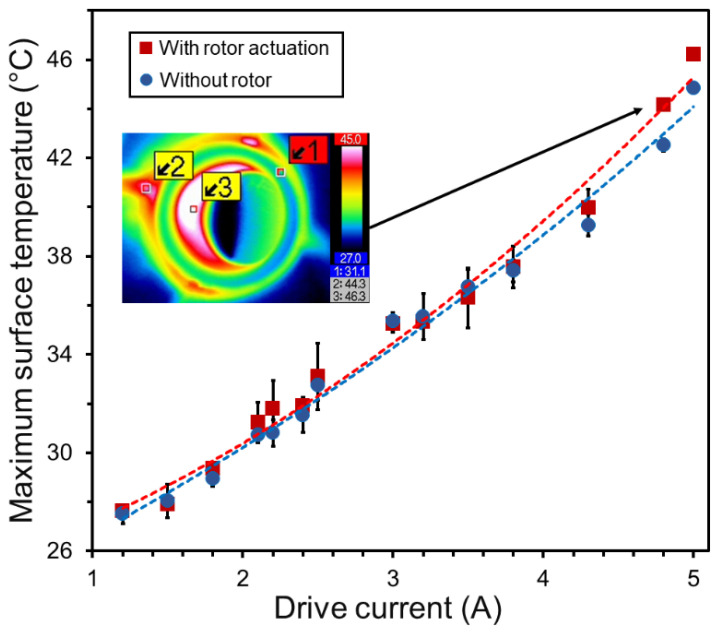
Maximum exterior surface temperature of the device (corresponding to the location with marker 2 shown in the inset infrared top-view image of the operating device) measured with varying drive currents for two cases, with the spinning rotor and without the rotor placed in the device. Markers 1, 2, and 3 in the infrared image (captured at a 4.8-A current) refers to the housing’s top ridge, exterior, and interior temperatures, respectively.

A comparison between the results with and without the rotating rotor plotted in Figure 8 indicates that, while they exhibited similar dependences, the case with the rotor showed slightly higher temperatures that became more apparent when the current exceeded ~3 A. This observation suggests two conditions—(1) the source of device heating is mostly Joule heating of the stator, and (2) there is another minor factor of heating that is caused by the rotor’s motion. The second condition is likely related to dynamic friction between the rotor and its cone base. The result with the rotating rotor indicates that at 4.8 A and 1400 rpm speed, the highest temperature reached 44.2 °C, suggesting that a current of 5 A is an approximate threshold level in the current prototype design. It should be noted however that the surface temperature of the scanner body itself was substantially (more than 10 °C) lower than the plotted maximum temperature on the power lead. Operation beyond 1500 rpm may still be feasible if the stator and its leads are properly packaged within the housing and additional thermal insulation layer (potentially with a trade-off of an increased outer diameter of the device).

### 4.4. Preliminary Optical Test

**Figure 9:**
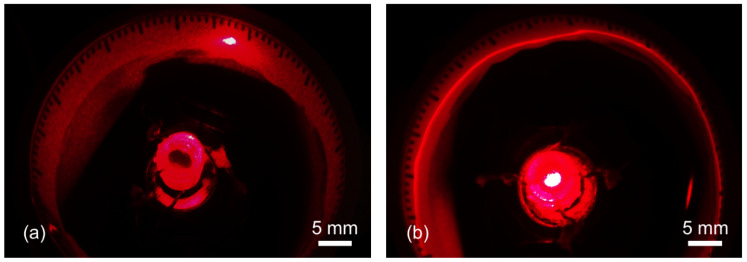
Optical scanning tests utilizing the fabricated prototype coupled with a laser source, demonstrating (**a**) stepwise scanning and (**b**) continuous rotation of the laser beam at 700 rpm.

A proof-of-concept scanning test was conducted to assess the device’s function for the circumferential rotation of an optical beam, the fundamental ability as an endoscopic distal scanner (Figure 9). In this test, a laser beam (650-nm wavelength, 1-mW power) was used as the optical source that was coupled with the scanner prototype. The laser beam was aligned to pass through the hollow structure of the scanner and hit on the back of the rotor near its perimeter region, bending it for circumferential scanning with the driven rotor. The scanned laser spot was imaged on the ring-shaped screen (40-mm diameter, graduated with 3° increments) and recorded using a digital video camera (Moticam 10, Motic, BC, Canada) during both stepping and high-speed mode operations. During the test, the scanner was driven with the high-speed mode at 700 rpm with 2.4 A of drive current. The prototype successfully rotated the laser beam 360° continuously in both modes (Figure 9a and Figure 9b, respectively) without any blind spot, as designed, showing the scanned laser trace as uniform as the case of the preceding ferrofluid-based device that rotated a prism mirror for laser scanning [35]. These results indicate the effectiveness of the scanner concept and operation method adopted in the current study.

## 5. Discussion

The experimental results demonstrated the concept of the proposed endoscopic distal scanner. While this scanner’s design significantly differs from the previous planar counterparts in terms of various factors, including the spatial relationship and interface between the rotor and stator as well as the optical components used, the speed-current trend appears similar to those of the previous devices [35,37]. This is presumably because the present actuator design leverages the electromagnetic architecture based on the ring stator and disc magnet rotor similar to them. The present design achieved up to 1500 rpm of rotation speed while maintaining stable operation of both the rotor and the drive circuitry. The electromechanical efficiency in output speed vs. input current for this design (Figure 7b) was found to be similar to those of the preceding ferrofluid-based scanners [35,39], but lower than those of their improved designs [36,37]. This might be related to two primary factors. One is friction. As described (Section 4.2), dynamic friction might have made nonnegligible negative impact on the output speed when it exceeded ~1000 rpm due to higher electromagnetic forces pulling the rotor more onto its cone base. This hypothesis is consistent with the thermal observation of the device (Section 4.3), which showed a slight temperature rise when the rotor was driven (compared with the case without the rotor) beyond the above speed level, where dynamic friction could have produced detectable minor heating on top of the stator’s Joule heating. The other likely factor is related to the distance between the rotor and the ring stator. It was previously shown that a reduction in the distance between the stator and rotor improved the output speed (given a local field strength being inversely proportional to the distance) [36,37]. In the present device, the average distance (from the center of the inclined rotor to the stator plane) is 2.16 mm, which is substantially greater than the corresponding distances used in the improved preceding designs (0.825 mm [36] and 0.7 mm [37]). Additionally, the magnet rotor used in this design is relatively larger than those designs (e.g., 4× greater mass compared with the one used in [37]), contributing to the larger inertia and thus requiring more torque and current to move the rotor.

An ability to operate at higher speeds is required for certain imaging modalities such as OCT, where high-speed scanning enables rapid image acquisition with a wider FOV, and is a key ability for functional imaging such as Doppler flow and OCT angiography that use repeated scan protocols [13]. The maximum speed for a given drive current is expected to increase by downsizing the rotor-stator combination including their spatial separation and minimizing the overall design (from its current diameter and height of both approximately 7 mm). This direction will also help reduce the drive current and hence power consumption or heating effects involved in a certain output speed.

The scanner prototype was successfully demonstrated to show 360° laser scanning in both stepping and high-speed modes without causing any shadow, due to the hollow design of the scanner, an important step toward further development for applications in multimodal imaging including OCT and RS. This newly developed actuation/scanning principle based on the precessing disc rotor architecture enabled significantly simplified scanner design by removing the need for additional mechanical and optical components. This experimentally verified design merit is expected to allow for drastic miniaturization in its length (to < a few millimeters), offering significantly higher tip-flexibility and maneuverability of the integrated endoscopic probes compared with, e.g., the major counterparts that used commercial small motors as the scanners whose lengths involved the centimeter range [28,31,32]. Moreover, the removal of fluid bearing from the architecture will not only ease its production process but also enhance the long-term reliability compared to the reported ferrofluid-based scanners [35,36,37,39]. This study revealed that the influence of friction or any other damping factor was negligible in achieving output speeds below 1000 rpm. This influence with higher speeds could be addressed or mitigated by way of minimizing friction, where the most straightforward approach will be to reduce the contact area between the rotor and its base via downsizing the disc rotor and the housing components. Additional potential measures include surface modification of the cone base of the rotor, e.g., smoothening the cone surfaces post 3D printing and/or depositing dry lubrication film [40,41] onto the cone surfaces. The development to follow will also include refining the device’s optical design and functionality. For example, the housing design with a 60° rotor angle (to prevent the bent laser beam from being interrupted by the outer walls of the housing) will be optimized to approach the ideal 45° angle for maximizing collection of reflected light from surrounding tissue in real application. The selection of an optimal backing mirror component (and potentially its protective layer) is another topic in this category. Further, the scanner device will be equipped with a protective cap and optical window to facilitate rotation and movement in all directions, toward the integration with fiber optics for endoscopic imaging/analysis tests.

## 6. Conclusions

This study has investigated an electromagnetic rotary actuator that leverages a precessional rolling movement of the magnetic rotor with a reflective surface, targeting the application to distal optical scanners for side-viewing endoscopy. The inclined rotor/mirror architecture in combination with the planar ring stator was revealed to effectively function as an optical scanner for both stepping and continuous high-speed rotations using the first experimental model. This design drastically simplified the scanner configuration by removing the need for extra optical and mechanical components, potentially allowing for significant axial downsizing and thus enhancing the flexibility of the scanner-integrated endoscopes. The developed prototype was tested to analyze its dynamic characteristics, including the stepping accuracy, electromechanical performance, and thermal behaviors. Proof-of-concept optical testing verified that a probing light could be scanned to provide shadow-less 360° FOV. This work paved the way to further development of the scanner device in order to achieve its compatibility with different endoscopic imaging modalities, such as OCT and RS, by leveraging the device’s ability for angular to continuous high-speed scanning. Future work will focus on optimization and miniaturization of the scanner design for integration with endoscopic probes, as well as their evaluation through ex-/in-vivo tests, toward enhancing the performance and potential applications of medical endoscope technology.

## Data Availability

The original contributions presented in this study are included in the article. Further inquiries can be directed to the corresponding author.
